# Clinical characteristics and prognostic model for extensive‐stage small cell lung cancer: A retrospective study over an 8‐year period

**DOI:** 10.1111/1759-7714.14289

**Published:** 2021-12-30

**Authors:** Jun Ni, Xiaotong Zhang, Hanping Wang, Xiaoyan Si, Yan Xu, Jing Zhao, Minjiang Chen, Li Zhang, Mengzhao Wang

**Affiliations:** ^1^ Department of Pulmonary and Critical Care Medicine, Peking Union Medical Hospital Chinese Academy of Medical Science & Peking Union Medical College Beijing China

**Keywords:** clinical characteristics, Cox regression analysis, extensive‐stage SCLC, nomogram, risk factors

## Abstract

**Background:**

Small cell lung cancer (SCLC) is a highly aggressive neuroendocrine tumor with a short replication time and a rapid growth rate. Prognostic factors for SCLC in clinical practice are scarce. Retrospective analysis of 8‐year extensive‐stage SCLC data from the Department Respiratory and Intensive Care Unit, Peking Union Medical College Hospital (Beijing, China) was performed to develop a risk prediction model that can facilitate the identification of extensive‐stage SCLC with differing prognosis in clinical practice.

**Methods:**

A retrospective analysis of data from patients with extensive‐stage SCLC at a single‐center from January 2013 to January 2021, including age, sex, ECOG physical score, immunohistochemistry (CgA, Syn, CD56, TTF1, and Ki67), staging, treatment regimen, laboratory examinations, and survival period, was performed. Clinical variables with potential prognostic significance were screened by univariate Cox analysis. Next, multifactor Cox risk prediction regression analysis was performed to establish an extensive‐stage SCLC risk prognostic model. Survival curves and ROC curves for high and low risk groups were plotted according to risk scores. Nomogram and calibration curves were developed to assess the accuracy of the risk prediction model.

**Results:**

This study included 300 patients who were diagnosed with extensive‐stage SCLC at our center from January 2013 to January 2021. The most common first presentation was respiratory symptoms, especially cough (162, 54%). The most common extra‐thoracic metastatic organs were bone (36.3%), liver (24.7%), brain (15.7%), and adrenal glands (15.7%). A total of 99% of patients received first‐line systemic therapy, with 86.3% of patients treated with platinum‐etoposide and 10.7% of patients treated with immune checkpoint inhibitor combined with platinum‐etoposide backbone. First‐line progression‐free survival was up to 198 days, and the median OS was 439 days. After Cox regression screening and backward stepwise selection, “time from initial therapy to relapse or progression (PFS1), liver metastases, adrenal metastases, M stage and first‐line treatment pattern” were retained to establish a prognostic model with an AUC value of 0.763. The prognostic model was shown as a nomogram with good agreement between predicted and observed outcomes.

**Conclusions:**

The first‐line treatment of SCLC patients admitted to our hospital in the past 8 years was relatively standardized, and the progression‐free survival and OS were slightly longer than those reported in the literature. We developed a prognostic risk score model for extensive‐stage SCLC to calculate individual survival in clinical practice.

## Graphical abstract

We developed a nomogram model for extensive‐stage small cell lung cancer to calculate individual survival. The results confirmed that a first‐line treatment paradigm of immune checkpoint inhibitors combined with chemotherapy prolongs overall survival.

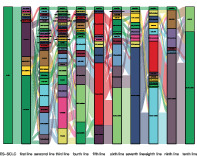

## INTRODUCTION

According to published cancer statistics, there were 235 660 new cases of lung cancer and 131 880 deaths worldwide in 2021, ranking it first in incidence and mortality among malignant tumors.^1^ Small cell lung cancer (SCLC) accounts for 10%–15% of lung cancers, with approximately 35 000 new cases per year.^1^, ^2^ Small cell lung cancer is characterized by aggressiveness, a high proliferative rate, and early metastasis, and 60%–70% of patients are already in extensive‐stage disease once diagnosed, with a median survival of only 8–12 months and 2‐year survival of only 5%–8%.^1^, ^3^, ^4^

In the traditional therapeutic field, platinum‐etoposide and thoracic radiotherapy are the main treatments for extensive‐stage SCLC.^5^, ^6^, ^7^ The efficiency of initial systemic antitumor therapy for extensive‐stage SCLC is 60%–70%, but almost all patients with extensive‐stage disease develop drug resistance or relapse within 1 year, and the efficiency of subsequent systemic therapy is less responsive.^8^, ^9^ Therefore, achieving sustained remission and long survival is rare for extensive‐stage SCLC with a median overall survival of only 8–10 months.^10^

With the advent of immunotherapy, the standard therapy changed following a randomized phase 3 trial (IMpower133) that demonstrated improved overall survival (OS) with the addition of atezolizumab, a PD‐L1 targeted immune checkpoint inhibitor, to platinum plus etoposide.^11^ In this study, the median OS was significantly longer with the addition of atezolizumab (12.3 months [95% CI: 10.8–15.9] vs. 10.3 months [95% CI: 9.3–11.3]). Subsequently, a randomized phase III clinical trial (CASPIAN) of durvalumab, another PD‐L1 targeted immune checkpoint inhibitor, plus platinum‐etoposide, showed sustained improvement of OS (12.9 months [95% CI: 11.3–14.7] vs. 10.5 months [95% CI: 0.3–11.2]).^12^, ^13^ The benefits of adding either of these anti‐PDL1 monoclonal antibodies to a standard platinum‐etoposide backbone are less evident in the median OS (2 months extension) but leads to an approximate doubling of the 2‐year survival rate (11%–22%). That is to say, a small subset of extensive‐stage SCLC achieves sustained remission.

Statistical prediction models are widely used among patients with cancer to predict outcomes. In the past decade, four nomograms in SCLC were built by Xie et al.^14^ Pan et al.^15^ Xiao et al.^16^ and Wang et al.^17^ with valuable prognostic data for clinicians. Because these models were published between 2015^14^ and 2018,^17^ immunotherapy combined with chemotherapy could not be used as a variable. Moreover, three of the studies^15^, ^16^, ^17^ were without further stratification for extensive‐stage SCLC, and two studies^16^, ^17^ lacked laboratory results, such as neutrophil‐to‐lymphocyte ratio and platelet‐to‐lymphocyte ratio.

In this study, we collected the clinical data of patients with extensive‐stage SCLC who were hospitalized at the Department of Respiratory and Intensive Care Unit at Peking Union Medical College Hospital from 2013 to 2021. We analyzed their demographic data, staging, and treatment patterns, and reviewed and summarized the disease characteristics at our single center. Finally, we explored the prognostic factors and developed a prognostic model.

## METHODS

### Data collection

The study was performed at the Department Respiratory and Intensive Care Unit, Peking Union Medical College Hospital (Beijing, China). We retrospectively reviewed the electronic medical records of all patients diagnosed with extensive‐stage SCLC from January 2013 to February 2021. The data that were collected comprised patient demographics (age, sex), ECOG physical score, initial manifestations, pathological immunohistochemistry (thyroid transcription factor‐1, neural cell adhesion molecule, chromogranin A, synaptophysin, Ki‐67%), laboratory examinations at baseline (white blood cell count, neutrophil count, lymphocyte count, red blood cell count, hemoglobin, platelet, neutrophil/lymphocyte ratio, platelet/lymphocyte ratio, serum potassium, serum natremia, albumin, alanine aminotransferase, total bilirubin, direct bilirubin, creatinine, urea, lactate dehydrogenase, creatine kinase, C‐reactive protein, ESR, ProGRP, and NSE), staging, metastasis, treatment regimens, and comorbidity.

Overall survival was defined as the length of time from diagnosis to death or last contact and used as the primary outcome. Progressive‐free survival (PFS) 1 was defined as the length of time from initial therapy to relapse or progression. TNM stage was defined according to the guidelines of American Joint Committee on Cancer (AJCC eighth edition).

The main inclusion criteria for patients in this study were as follows: (1) Patients with histopathological diagnosis of SCLC, (2) extensive‐stage disease, (3) at least one tumor assessment in those receiving systemic therapy, and (4) complete medical records. Exclusion criteria included nonpathologically confirmed SCLC, limited‐stage disease, or incomplete clinical information.

### Statistical analysis

Continuous variables are expressed as the mean ± standard deviation (SD). Clinicopathological features are expressed as median and interquartile (IQR). Categorical variables are represented by numbers and proportions (%). For missing data, multiple imputation was performed using the chain equation package (MICE) of R software (version 3.6.1).

With OS and status as dependent variables, univariate and multivariate Cox regression analyses were used to assess prognostic factors, including clinicopathological and pretreatment hematological markers. Variables identified by multifactorial Cox regression analysis (*p* < 0.01) were selected as risk factors. After these analyses, a risk prediction model was formed in which the risk score for each sample was calculated according to the following formula:

$$\text{Risk score}=\sum \text{variable expression level}\;\left(i\right)*\text{coeffective}\;\left(i\right)$$

According to the risk score, the difference in OS between the high‐ and low‐risk groups and the difference in OS between the high and low risk of different clinical variables were calculated. The performance of the prediction model was assessed as the area under the receiver operating characteristic (ROC) curve. A nomogram was built using the regression modeling strategies (rms) package of R (version 3.6.1). The performance of the nomogram was assessed using the consistency index (C‐index) and calibration curves.

## RESULTS

### Patient characteristics and treatment patterns

In total, 300 patients with extensive‐stage SCLC were included in this study, including 241 men (80.3%) with a median age of 64 years (21–91 years). Two hundred and twenty‐five patients had a history of smoking, with a mean smoking index of 32 ± 29 pack‐years, and 75 patients (25%) had never smoked. Cardiovascular disease (136, 43.5%), endocrine disease (71, 23.7%), and respiratory disease (52, 17.3%) were the most common comorbidities. Twelve (4%) of the enrolled patients had immediate family members who had suffered from lung cancer. Two hundred and two patients were admitted as outpatients with respiratory symptoms or other initial symptoms, including bone pain (10%) and superior vena cava syndrome (2.3%), and approximately 30 asymptomatic cases were identified by physical examination. Neuroendocrine markers, including chromogranin (75.7%), synaptophysin (90.4%), and neural cell adhesion molecule (71.8%), were the most sensitive. The proportion of patients with Ki‐67 index greater than 80% was 74.7%. Transcription termination factor 1 was positive in 90.4% extensive‐stage disease. According to TNM classification, more than 60% patients were T4‐N3‐M1. Among them, 11% cases were associated with paraneoplastic syndromes. Common paraneoplastic syndromes included inappropriate antidiuretic hormone and Cushing’s syndrome (10%), Lambert‐Eaton syndrome (0.3%), encephalomyelitis (0.3%), and peripheral neuropathy (0.3%). Bone metastasis was the most common metastatic site in 109 cases (36.3%), followed by liver (24.7%), brain (16.3%), adrenal (15.7%), abdominal lymph nodes (4%), pancreas (2%), spleen (1%), bone marrow (1%), and meninges (0.3%). The patient characteristics are summarized in Table 1.

**TABLE 1:** Clinical characteristics of extensive‐stage SCLC and univariate analysis

Characteristics	Total (*n* = 300)	*p*‐value^a^ (95% CI)
Demographics		
Age, median (IQR)	64 (56–70)	0.013 (1.004–1.032)
Male sex, no. (%)	241 (80.3)	0.521 (0.806–1.531)
Smoking index (packs/year)		0.877 (0.996–1.005)
Mean ± SD	32 ± 29	
Median (IQR)	30 (1.25–50)	
ECOG physical scores，no (%)		0.029 (1.039–2.009)
0–1	247 (82.3)	
≥2	53 (17.7)	
Comorbid conditions, yes/no (%)	215 (71.7%)	0.912 (0.766–1.348)
Digestive	37 (12.3%)	
Cardiovascular	136 (45.3%)	
Endocrine	71 (23.7%)	
Cerebrovascular	15 (5%)	
Respiratory	52 (17.3%)	
Others	27 (9%)	
Tumor history, no (%)		0.071 (0.978–1.710)
Lung cancer	12 (4%)	
Others	12 (4%)	
Immunohistochemistry, no (%)		
CgA positive	228 (75.7%)	0.144 (0.615–1.073)
Syn positive	275 (91.4%)	0.057 (0.452–1.012)
CD56 positive	216 (71.8%)	0.038 (0.588–0.985)
TTF‐1 positive	272 (90.4%)	0.697 (0.576–1.447)
Ki‐67% ≥ 80%	224 (74.7%)	0.206 (0.897–1.657)
Manifestations, yes/no (%)	259 (86.3%)	0.457 (0.793–1.674)
Cough	162 (54%)	
Hemoptysis	10 (3.3%)	
Dyspnea	30 (10%)	
Bone pain	30 (10%)	
Superior vena cava syndrome	7 (2.3%)	
Hoarseness	1 (0.3%)	
Others	37 (12%)	
Staging, no (%)		
T	T1 20 (6.6%)	0.962 (0.405–2.363)
	T2 39 (13.0%)	0.421 (0.635–2.968)
	T3 32 (10.6%)	0.819 (0.499–2.410)
	T4 198 (65.8%)	0.804 (0.537–2.232)
	Tx 11 (3.7%)	Reference
N	N0 6 (2%)	0.894 (0.173–4.635)
	N1 8 (2.7%)	0.150 (0.044–1.610)
	N2 86 (28.6%)	0.975 (0.249–4.194)
	N3 196 (65.1%)	0.722 (0.319–5.217)
	Nx 5 (1.3%)	Reference
M	M0 30 (10%)	0.003 (1.256–3.166)
	M1 270 (90%)	
Metastatic sites, no (%)		
Hepatic metastasis	74 (24.7%)	0.001 (1.344–2.421)
brain metastasis	49 (16.3%)	0.033 (1.028–2.009)
Bone metastasis	109 (36.3%)	0.015 (1.065–1.803)
Adrenal metastasis	47 (15.7%)	0.001 (1.522–2.953)
Others	43 (14.3%)	0.974 (0.951–1.050)
Paraneoplastic syndrome, yes/no (%)	37 (12.3%)	0.288 (0.844–1.768)
Inappropriate ADH secretion	34 (11.3%)	
Lambert‐Eaton myasthenic syndrome	1 (0.3%)	
Encephalomyelitis	1 (0.3%)	
Peripheral neuropathy	1 (0.3%)	
Laboratory data (baseline) mean ± SD		
WBC (×10^9^/l)	7.2492 ± 2.39	0.300 (0.899–1.033)
Neutrophils (×10^9^/l)	4.9450 ± 2.18	0.0496 (0.710–0.995)
Lymphocytes (×10^9^/l)	1.6262 ± 0.62	0.044 (0.710–0.995)
RBCs (×10^9^/l)	4.3745 ± 0.54	0.014 (0.525–0.930)
Hemoglobin (g/l)	134.2545 ± 16.42	0.013 (0.986–0.998)
Platelet (×10^9^/l)	244.8438 ± 85.73	0.118 (0.998–1.000)
Neurophil to lymphocyte ratio	3.3208 ± 3.04	0.050 (0.999–1.085)
Platelet to lymphocyte ratio	172.0526 ± 100.28	0.161 (0.999–1.002)
Serum potassium (mmol/l)	3.9287 ± 0.42	0.686 (0.769–1.189)
Serum natremia (mmol/l)	136.4798 ± 6.02	0.339 (0.968–1.011)
Albumin (g/l)	139.6786 ± 4.05	0.572 (0.965–1.020)
Alanine aminotransferase (U/l)	27.5804 ± 35.83	0.729 (0.997–1.004)
Total bilirubin (μmol/l)	11.7484 ± 6.54	0.235 (0.991–1.036)
Direct bilirubin (μmol/l)	3.5054 ± 3.71	0.110 (0.992–1.079)
Creatinine (μmol/l)	80.4688 ± 14.96	0.777 (0.987–1.009)
Urea (mmol/l)	5.1646 ± 1.61	0.656 (0.931–1.119)
Lactate dehydrogenase (U/l)	276.9277 ± 215.70	0.035 (1.000–1.001)
ProGRP (pg/ml)	1071.1806 ± 1949.34	0.816 (0.997–1.001)
NSE (ng/ml)	61.2883 ± 26.32	0.323 (0.997–1.001)
First treatment pattern, no. (%) (chemo vs. ICI + chemo)	297 (99%)	0.0025 (0.214–0.719)
Chemotherapy	265 (88.3%)	
Carboplatin‐etoposide	212 (70.6%)	
Cisplatin‐etoposide	46 (15.3%)	
Etoposide	5 (1.7%)	
Others	2 (0.7%)	
ICI plus chemotherapy	32 (10.7%)	
PD‐L1‐platinum‐etoposide	14 (4.7%)	
PD1‐platinum‐etoposide	4 (1.3%)	
PD1‐platinum‐irinotecan	10 (3.3%)	
CTLA4‐platinum‐etoposide	4 (1.3%)	
First chest radiation, yes/no (%)	133 (44.3%)	0.019 (0.566–0.951)
Prophylactic cranial irradiation, yes/no (%)	12 (4%)	0.280 (0.389–1.313)
Survival (day), median (IQR)		
PFS1	198 (93–283)	0.001 (0.997–0.999)
OS	439 (246–730)	

*Abbreviations*: ADH, antidiuretic hormone; CD56, neural cell adhesion molecule; CgA, chromogranin A; ESR, erythrocyte sedimentation rate; ICI, immune checkpoint inhibitor; IQR, interquartile; NES, neuron specific enolase; OS, time from diagnosis to death or last contact; PFS1, time from initial therapy to relapse or progression; ProGRP, pro‐gastrin releseasing peptide; RBC, red blood cell; Syn, synaptophysin; TTF‐1, thyroid transcription factor‐1; WBC, white blood cell.

^a^ Univariate analysis of the cohort.

**FIGURE 1:**
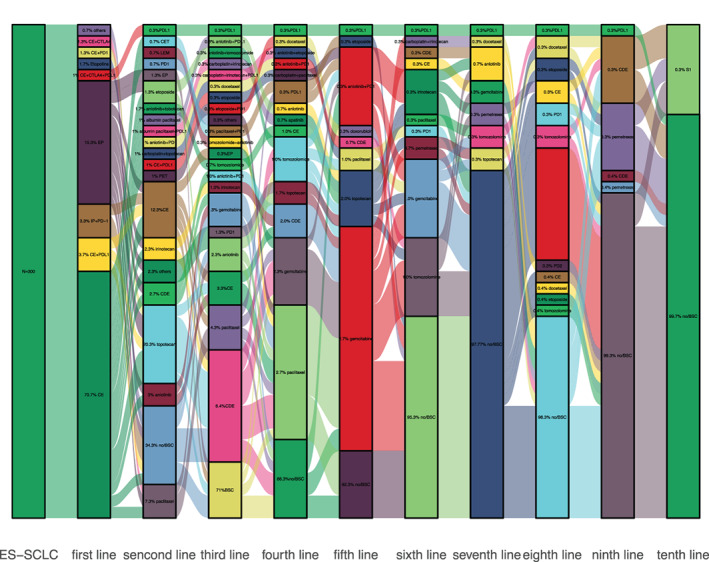
Treatment patterns of extensive‐stage SCLC. BSC, best supportive care; CE, carboplatin‐etoposide; EP, cisplatin‐etoposide; CVA, cyclophosphamide‐doxorubicin‐vincristine; PET, platinum‐etoposide‐topotecan

Regarding treatment regimens, 296 (98.6%) patients received first‐line systemic therapy (three patients died without palliative care) with a median PFS of 198 days (18–1459 days), 187 patients (62.3%) entered second‐line treatment with a median PFS of 105 days (9–2049 days) and continued chemotherapy for primary disease in 14.3% (43 cases), 90 patients (30%) received third‐line treatment with a median PFS of 83 days (9–438 days), 45 patients (15%) received fourth‐line treatment with a median of 95 days (11–415 days), and 23 patients (7.6%) received fifth‐line treatment with a median of 78 days (41–243 days). In initial therapy, 212 patients received carboplatin‐etoposide, 46 received cisplatin‐etoposide, and 22 received immune checkpoint inhibitor plus platinum‐etoposide. Because of a clinical trial at our center, 10 patients received irinotecan‐platinum plus PD‐1 targeted immune checkpoint inhibitor. The treatment pattern is visualized by Sankey diagram in Figure 1.

### Extensive‐stage small cell lung cancer prognosis model

To avoid statistical bias and degradation of performance due to missing values, the multiple interpolation of the chain equation (MICE) package in R software was applied to achieve multiple interpolation.

#### Univariate Cox risk regression analysis

Univariate analysis was used to identify the parameters that were significantly associated with OS. As shown in Table 1, PFS1, liver metastases, adrenal metastases, and M stage were significantly associated with OS (*p* < 0.01). The treatment pattern of first‐line therapy also significantly impacted on OS (*p* < 0.01).

#### Lasso regression

**FIGURE 2:**
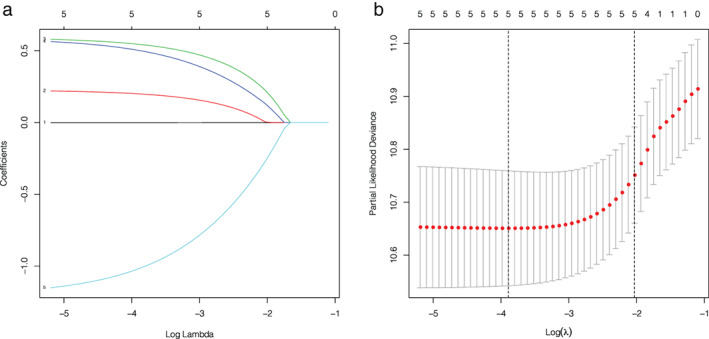
(a) LASSO coefficient curve for five independent variables. (b) Logarithmic sequence coefficient distribution

To avoid overfitting, Lasso regression was performed (R software glmnet package). The lasso coefficient curves for the five independent variables are shown in Figure 2(a). The adjustment parameter (lambda) in the lasso regression was chosen as the minimum criteria for 10‐fold cross‐validation. Figure 2(b) shows the distribution of coefficients for a logarithmic (lambda) series. At the optimal Lambda.min, all five clinical variables had nonzero coefficients.

#### Multivariate Cox risk regression analysis

**FIGURE 3:**
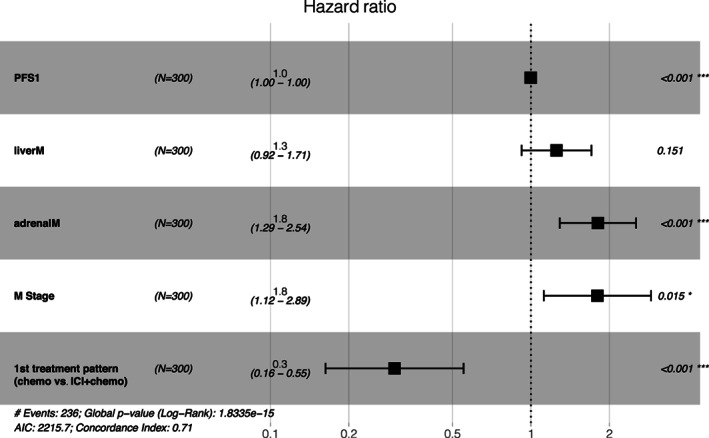
Multivariate Cox analysis forest plot (*p < 0.001)

In univariate analysis and Lasso regression, PSF1, liver metastases, adrenal metastases, M stage, and first‐line treatment pattern were significantly associated with OS. To further determine their prognostic significance, these five variables as risk factors were used to build the prognostic model. A forest plot is shown in Figure 3. The RS was calculated by the following formula:

$$\text{Risk score}=\left(-0.0021109\times \mathrm{PFS}\;\left[\text{year}\right]\right)+\left(0.22595064\times \text{liver metastases}*\right)+\left(0.5926505\times \text{adrenal metastases}*\right)+\left(0.58798716\times M\;\text{stage}\right)+\left(-1.2055895\times \text{first}-\text{line}\;{\text{treatment pattern}}^{△}\right)$$

*Point 1, existence metastases; point 0, no metastases; ^**△**^point 1, ICI combined with chemotherapy; point 0, chemotherapy.

#### Nomogram prognostic model

**FIGURE 4:**
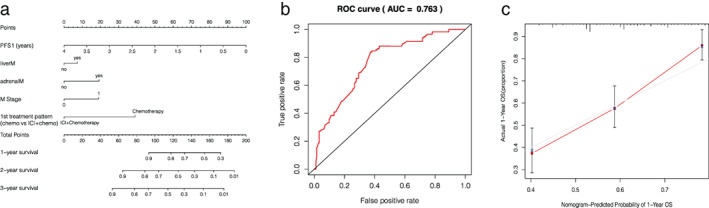
(a) Nomogram prognostic module and prediction of survival probability. LiverM, liver metastases; adrenal, adrenal metastases. (b) ROC curve analysis of the prognostic model for extensive‐stage SCLC. (c) Calibration curves comparing predicted and actual survival proportions at 1 year

All five risk factors were used to develop the nomogram. As shown in Figure 4(a), the nomogram predicted the 1‐, 2‐, and 3‐year OS ratio. The first‐line treatment pattern mainly contributed to prognosis, followed by adrenal metastases, liver metastases, M stage, and PFS1. The ROC curve of the nomogram model was plotted with an area under curve of 0.763 (Figure 4(b)), and the concordance index (C‐index) was 0.71. Calibration plots showed good agreement between predicted and observed 1‐year OS in our cohort (Figure 4(c)).

#### Survival analysis based on the nomogram

**FIGURE 5:**
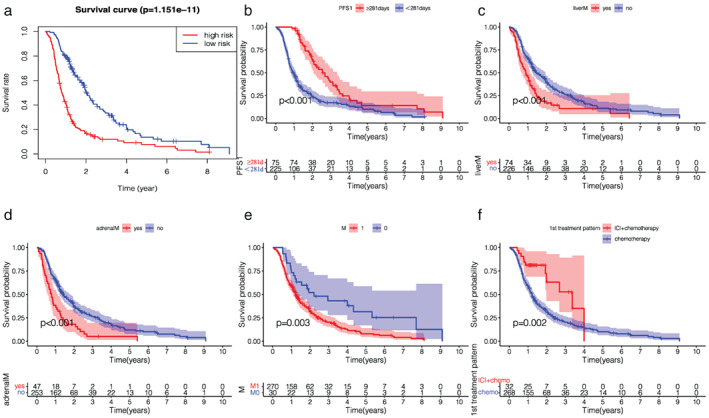
(a) Kaplan–Meier overall survival curve for high and low risk patients with extensive‐stage SCLC. (b) PFS1: Patients with PFS shorter than 281 days were in the high‐risk group. (c) liverM: Patients with liver metastases were in the high‐risk group. (d) adrenalM: Patients with adrenal metastases were in the high‐risk group. (e) M‐stage: Patients with M1 were in the high‐risk group. (f) First‐line treatment pattern: Patients who received first‐line chemotherapy were in the high‐risk group

According to the risk score for each patient in the model, patients were divided into high‐ and low‐risk groups. The Kaplan–Meier survival curves of high‐ and low‐risk patients are shown in Figure 5.

## DISCUSSION

SCLC is a poorly differentiated highly malignant tumor originating from bronchial neuroendocrine cells. Two‐thirds of patients are found to have extensive‐stage disease and can only be treated with systemic palliative therapy, with a poor overall prognosis and a 2‐year survival rate of approximately 10%.^18^ The systemic treatment regimen for extensive‐stage SCLC is relatively poor, but immunotherapy has broken this deadlock. The IMpower‐133^11^, ^19^ and CASPIN^12^, ^13^ trials pioneered immune checkpoint inhibitors combined with chemotherapy in untreated extensive‐stage SCLC. Previous prognostic models for SCLC were mostly limited to conventional therapy (chemotherapy and radiotherapy),^14^, ^16^, ^17^ for which we included the first‐line treatment pattern (ICI + chemo vs. chemo) as a candidate variable. Overall survival is the ultimate indicator for evaluating therapeutic regimens; thus, we selected OS as the study endpoint on the basis of extensive‐stage SCLC data from our center over 8 years, and aimed to summarize the clinical characteristics of extensive‐stage SCLC and develop a clinical prediction model to effectively identify patients with different prognoses.

In this study, the clinical characteristics of 300 patients with extensive‐stage SCLC were consistent with previous studies.^3^, ^18^, ^20^, ^21^ Patients with extensive‐stage SCLC were predominantly smokers (75%) and male (80.3%), and the most common initial symptoms were mainly cough (54%), dyspnea (10%), bone pain (10%), asymptomatic (12%), and paraneoplastic syndromes (11%).^22^ The most common comorbidities were hypertension, diabetes mellitus,^23^ and chronic obstructive pulmonary disease,^24^ accounting for 33.7%, 19%, and 7% of cases, respectively. The most common extra‐thoracic metastatic organs were bone, liver, brain, and adrenal gland, at 36.3%, 24%, 7%, 16.3%, and 15.7%, respectively.^25^ The first‐line regimen was platinum combined with etoposide in 86.3% of cases, with a median first‐line PFS of 198 days and a median OS of 439 days. The median survival was slightly prolonged compared with large‐scale clinical studies,^26^ considering some correlations with ethnic differences.^17^

In this study, a prognostic model was developed based on clinical and laboratory data from a single center of 300 patients with extensive‐stage SCLC. This model incorporates five risk factors, PFS1, liver metastasis, adrenal metastasis, M‐stage, and first‐line treatment regimen, to predict the probability of survival in individual extensive‐stage SCLC and aid individualized treatment selection.

The nomogram calculates the probability of survival for an individual rather than simply matching risk groups (Manchester Score^27^ and Spain prognostic index^28^) and is considered a more effective prognostic predictor. In contrast to the previously published nomogram, this model only includes patients with extensive‐stage SCLC and includes first‐line immunotherapy, facilitating the choice of immune checkpoint inhibitor therapy for patients with extensive‐stage SCLC. Meanwhile, the model had an ROC‐AUC score of 0.736 and a C‐index of 0.71, indicating a strong predictive power. Compared with the results of Zhong et al.^29^ based on the SEER program, the candidate variables of this nomogram model included laboratory data, and the ethnic background of all patients was Asian, which was more clinically meaningful.

In this nomogram model, multivariate risk regression analysis, PFS1, liver metastases, adrenal metastases, and first‐line treatment regimen were retained as risk factors. As shown in previous studies,^10^ the OS of patients with extensive‐stage SCLC is largely dependent on the effective duration of the initial treatment, and as the PFS by initial treatment increased, the OS of the patient increased. Three metastasis‐related variables—M stage, liver metastasis, and adrenal metastasis—were included in this model. Consistent with the model developed by Wang et al.^17^ Pan et al.^15^ and Xie et al.^14^ the presence of distant metastases was associated with poor prognosis. Xiao et al.^16^ only discussed SCLC Veterans Administration Staging without further inclusion of AJCC TNM staging. Moreover, this model focused on the prognostic implications of differences in metastatic organs, with liver metastases and adrenal metastases potentially presenting life‐threatening or significant clinical symptoms.

This model proposes that immune‐combination chemotherapy in first‐line treatment of extensive‐stage SCLC may improve OS, which is consistent with the results of the IMpower 133^11^, ^19^ and CASPIAN trials.^12^, ^13^ Four previous nomogram models^14^, ^15^, ^16^, ^17^ have suggested a correlation between differences in treatment regimens and OS, but none involved immune checkpoint inhibitor‐combination chemotherapy. However, clinical treatment decisions need to be individualized according to the patient, and this model only provides a convenient strategic reference for predicting survival.

Laboratory tests, including NSE, ProGRP, LDH, serum sodium, neutrophil‐to‐lymphocyte ratio, and platelet‐to‐lymphocyte ratio, were included as candidate variables in this model, but univariate regression analysis showed that these laboratory variables were not significantly correlated with OS (*p* > 0.01). Therefore, these variables were not included as risk factors in the final nomogram model, considering the fact that only patients with extensive‐stage disease and not all SCLC patients were included in this study.

This study had several limitations. First, this prognostic model was built according to OS, and there may be potential confounding effects arising from noncancer‐specific deaths and unknown complications. Second, this model requires further validation in other extensive‐stage SCLC cohorts to improve the reliability of the prognostic model.

## CONFLICT OF INTEREST

All authors declare no conflict of interest.
