# Corrigendum

**DOI:** 10.1111/1759-7714.14585

**Published:** 2022-08-14

In Ni *et a*l.^1^ the following error was published on page 539.

The authors would like to correct their affiliation and correspondence address, whereas, ‘Peking Union Medical Hospital’ should read as ‘Peking Union Medical College Hospital’.

The affiliation and correspondence section should read as below:

**Affiliation**

Department of Pulmonary and Critical Care

Medicine, Peking Union Medical College Hospital,

Chinese Academy of Medical Science & Peking

Union Medical College, Beijing, China

**Correspondence**

Li Zhang and Mengzhao Wang, Department of

Pulmonary and Critical Care Medicine, Peking

Union Medical College Hospital, Chinese Academy of

Medical Science & Peking Union Medical College,

Beijing 100730, China.

The authors apologize for the error and any inconvenience it may have caused.
